# Evaluation of a novel West Nile virus transmission control strategy that targets *Culex tarsalis* with endectocide-containing blood meals

**DOI:** 10.1371/journal.pntd.0007210

**Published:** 2019-03-07

**Authors:** Chilinh Nguyen, Meg Gray, Timothy A. Burton, Soleil L. Foy, John R. Foster, Alex Lazr Gendernalik, Claudia Rückert, Haoues Alout, Michael C. Young, Broox Boze, Gregory D. Ebel, Brady Clapsaddle, Brian D. Foy

**Affiliations:** 1 Arthropod-borne and Infectious Diseases Laboratory, Department of Microbiology, Immunology and Pathology, Colorado State University, Fort Collins, CO, United States of America; 2 UMR ASTRE, INRA-CIRAD, Montpellier, France; 3 Vector Disease Control International, Little Rock, AR, United States of America; 4 TDA Research, Inc., Wheat Ridge, CO, United States of America; The Connecticut Agricultural Experiment Station, UNITED STATES

## Abstract

Control of arbovirus transmission remains focused on vector control through application of insecticides directly to the environment. However, these insecticide applications are often reactive interventions that can be poorly-targeted, inadequate for localized control during outbreaks, and opposed due to environmental and toxicity concerns. In this study, we developed endectocide-treated feed as a systemic endectocide for birds to target blood feeding *Culex tarsalis*, the primary West Nile virus (WNV) bridge vector in the western United States, and conducted preliminary tests on the effects of deploying this feed in the field. In lab tests, ivermectin (IVM) was the most effective endectocide tested against *Cx*. *tarsalis* and WNV-infection did not influence mosquito mortality from IVM. Chickens and wild Eurasian collared doves exhibited no signs of toxicity when fed solely on bird feed treated with concentrations up to 200 mg IVM/kg of diet, and significantly more *Cx*. *tarsalis* that blood fed on these birds died (greater than 80% mortality) compared to controls (less than 25% mortality). Mosquito mortality following blood feeding correlated with IVM serum concentrations at the time of blood feeding, which dropped rapidly after the withdrawal of treated feed. Preliminary field testing over one WNV season in Fort Collins, Colorado demonstrated that nearly all birds captured around treated bird feeders had detectable levels of IVM in their blood. However, entomological data showed that WNV transmission was non-significantly reduced around treated bird feeders. With further development, deployment of ivermectin-treated bird feed might be an effective, localized WNV transmission control tool.

## Author summary

West Nile virus (WNV) is a mosquito-borne virus that causes significant disease and death every year in humans, domesticated animals, and wildlife. Control of WNV transmission is focused on controlling the mosquito vector through applications of insecticides directly to the environment. In this study, we evaluate a novel control strategy for WNV transmission by targeting the main mosquito bridge vector in the Great Plains region, *Culex tarsalis*, through its blood feeding behavior. Because *Culex tarsalis* favor taking blood meals from particular bird species, our strategy aims to target these bird species with endectocide-treated bird feed that will result in lethal blood meals for *Cx*. *tarsalis*. In this study, we developed a safe and effective formulation of ivermectin-treated diet that resulted in increased mortality for *Cx*. *tarsalis* blood fed on birds consuming this treated diet as compared to mosquitoes feeding on control birds. We also conducted a pilot field trial in Fort Collins, Colorado to test this strategy in a natural transmission cycle, which demonstrated promising results.

## Introduction

West Nile virus (WNV) is an arthropod-borne flavivirus, and the leading cause of domestically acquired arboviral disease in the United States [1,2], resulting in significant disease and death every year in humans, domesticated animals, and wildlife. From 1999–2017, >48,000 cases of human WNV disease and >2000 deaths were reported to the CDC [3], but the total number of individuals in the U.S. who have been made ill from WNV is estimated to be greater than 1 million, or approximately 1 of every 5 persons infected (>5 million infected individuals) [4]. Control of WNV transmission remains focused on vector control through larvicide and adulticide applications [5]. Larvicide applications are generally preferred to adulticide applications as they are more cost-effective and less environmentally-damaging due to more direct and efficient targeting of mosquitoes [6,7]. While previous studies have demonstrated the effectiveness of larvicide applications to catch basins, a common *Culex* larval habitat, in reducing the number of mosquitoes [8,9], the efficacy may vary significantly with suboptimal catch basin design or environmental conditions [10,11]. Aerial spraying can be costly [12], but is effective in reducing target mosquito populations [13–16], and has been linked to reductions in human WNV cases in a treated area relative to an untreated area [15] and in entomological measures of WNV risk [16]. Similar ground ultra-low volume application of adulticides may reduce target mosquito populations under ideal conditions, but studies have provided inconclusive data on their effect on WNV infection rates in mosquitoes or subsequent virus transmission [17–20]. Additionally, off-target effects can occur despite optimal calibration of adulticide applications to host-seeking and active times for target vector species [21–23]. Insecticide applications also often face community opposition due to environmental and toxicity/allergenicity concerns [24–28] and are often restricted to urban and semi-urban communities that can afford to fund them [29,30].

WNV is maintained in an enzootic cycle between *Culex* mosquitoes and avian hosts. The highest WNV disease incidence occurs along the Great Plains region of the United States [31], as the irrigated agriculture provides a supportive habitat for the main WNV bridge vector of the region, *Culex tarsalis* [32].Therefore, blood meals by *Cx*. *tarsalis* from often-bitten avian species may be utilized to selectively target adult females through their blood feeding behavior. Given that the majority of *Cx*. *tarsalis* blood meals on the northern Colorado plains may come from select species during the WNV transmission season [33], effective targeting of these preferred hosts with endectocide-treated bird feed could result in control of WNV transmission.

Previous studies have assessed the use of systemic endectocides provided to wild animals to control tick vector populations. Pound et al. evaluated ivermectin (IVM)-treated corn that was fed to white-tailed deer (*Odocoileus virginianus*) in a treatment pasture to control tick populations [34]. *Amblyomma americanum* collections from treatment pastures showed a 83.4% reduction in adults, 92.4% in nymphs, and 100.0% in larvae compared to control pastures [34]. IVM-treated feed provided to *O*. *virginianus*, which is the definitive host for the reproductive stage of *Ixodes scapularis*, has also been explored as a method for controlling this vector of Lyme disease. Rand et al. provided an island community of white-tailed deer with IVM-treated corn for 5 consecutive spring and fall seasons [35]. A treatment effect was observed in island deer that reached target IVM sera concentrations resulting in reductions in adult tick density, engorgement, and oviposition rates as well as reduced rates of larval eclosion from any laid eggs compared to collections from untreated deer on a control island [35]. Dolan et al. also conducted a field study that targeted the rodent reservoirs of Lyme disease to reduce the infection prevalence of *Borrelia burgdorferi* and *Anaplasma phagocytophilum* with antibiotic-treated bait. Between treated and control areas, they found that *B*. *burgdorferi* prevalence was reduced by 87% and *A*. *phagocytophilum* by 74% in small mammals, and in questing nymphal ticks, *B*. *burgdorferi* prevalence was reduced by 94% and *A*. *phagocytophilum* by 92% [36]. A field study testing the passive application of topical acaricide during bait consumption showed reductions of 68% and 84% of nymphal and larval *I*. *scapularis* found on white-footed mice, accompanied by a 53% reduction in the *B*. *burgdorferi* infection rate of white-footed mice and a 77% decrease in the questing adult *I*. *scapularis* abundance between control and treated properties [37].

Rodent baits with feed-through and systemic insecticide activity have also been evaluated to control the phlebotomine sand fly vectors of zoonotic cutaneous leishmaniasis and visceral leishmaniasis. A wide variety of insecticides have been tested for efficacy against multiple phlebotomine sand fly species using larval and adult blood feeding bioassays in multiple rodents. Methoprene, pyriproxyfen, novularon, eprinomectin, ivermectin, and diflubenzuron have been tested for efficacy within the lab [38–41], while fipronil has been additionally tested in field studies [40,42,43]. Systemic insecticides have also been used to target plague transmission, where field trials have assessed imidalcloprid-treated bait for controlling flea populations in California ground squirrels (*Spermphilus beechyi)*, black-tailed prairie dogs *(Cynomys ludovicianus)*, and other rodents [44–46]. To our knowledge, this strategy of endectocide-treated baits has not been evaluated in birds for arbovirus control.

IVM use in birds is primarily off-label; however, IVM has been administered to treat multiple species of parasites that infest birds, including falcons, cockerels, and chickens [47–50]. Moreno et al. characterized the pharmacokinetics, metabolism, and tissue profiles of IVM in laying hens *(Gallus gallus)* with IVM delivered using intravenous (IV) and oral routes [51]. For both IV and oral routes, expected pharmacokinetic profiles and tissue distributions consistent for a highly lipophilic drug were observed [51]. Bennett et al. demonstrated transfer of IVM through crop milk when adult pigeon pairs were given 3.3 μg/mL IVM dosed in drinking water and housed with brooding squab, and IVM was subsequently detected in squabs following 3 days of daily adult pigeon IVM dosing [52].

In this present study, we evaluated endectocide-treated bird feed as a systemic endectocide to target *Cx*. *tarsalis*. 50% lethal concentrations for selamectin, eprinomectin, and ivermectin were determined in artificial blood meals. IVM-treated bird feed was evaluated for safety and consumption rates in chickens. Mosquitocidal effects in *Cx*. *tarsalis* fed on IVM-treated birds were also characterized. Lastly, we present the results of a pilot field trial conducted in Fort Collins, CO in 2017 that examined the safety of IVM-treated bird feed in the field and efficacy on entomological indices of WNV transmission.

## Methods

### Ethics statement

Animal research was done under CSU IACUC study protocol 16-6552A. Animal euthanasia was applied using sodium pentobarbital as approved in the IACUC study protocol. Field research was done under Colorado Parks and Wildlife Scientific Collection License #17TRb2104 and Fort Collins Natural Areas Permit #914–2017.

### Mosquito membrane feeding assays

*Cx*. *tarsalis* (Bakersfield colony) were reared in standard insectary conditions (28 ˚C, 16:8 light cycle). Approximately 150 larvae were reared in roughly 3 gallons of water and fed 2.5 grams of powdered Tetramin fish food daily until pupation. Adults were housed at approximately 300 per cage and fed *ad libitum* sugar and water until separated for bioassays. Mosquito bioassays were performed to determine the lethal concentrations resulting in 50% mortality (LC_50_) by adding drug (eprinomectin, selamectin, and IVM) into defibrinated calf blood (Colorado Serum Company) at serial dilutions for artificial membrane feeding. Following blood feeding, *Cx*. *tarsalis* were knocked down with CO_2_, and fully-engorged females were collected and held for 5 days in the same insectary conditions. For all bioassays, mosquito mortality was recorded every 24 hours and analyzed using Kaplan-Meier survival curves and compared using Mantel-Cox (log-rank) test. LC_50_ values were calculated using a nonlinear mixed model with probit analysis [53].

Artificial membrane blood feeds were also used to test the effects of IVM and WNV on *Cx*. *tarsalis* mortality. The WNV strain used was a 2012 Colorado isolate propagated in Vero cells. Negative controls were DMEM (Dulbecco’s Modified Eagle Media) and DMSO (dimethyl sulfoxide) at the same volumes as WNV and IVM, respectively. For the concurrent blood feed of WNV and IVM, IVM at 73.66 ng/mL (LC_75_) and WNV at low titer (5x10^5^ PFU/mL) or high titer (10^7^ PFU/mL) were fed in a membrane blood meal to *Cx*. *tarsalis* and mortality was observed as described above. For the WNV-exposure followed by an IVM blood feed, mosquitoes were fed a first blood meal containing 10^7^ PFU/mL of WNV or DMEM for a mock-exposure. Fully engorged females were sorted and held for 10 days, then fed a second blood meal containing 73.66 ng/mL IVM, after which fully blood fed females were sorted and mortality observed.

### Birds

4–6 weeks old white leghorn chickens were divided into groups (n = 4) that were housed separately, and which were provided clean water daily and control (untreated) diet consisting of a cracked corn mix (Chick Start and Grow, Northern Colorado Feeders Supply) mixed with any additives that were also added to IVM-treated diet for 3 or 7 consecutive days. IVM-treated diet consisted of two formulations: an Ivomec formulation where liquid Ivomec (Merial) was mixed directly into the cracked corn mix and a powder IVM formulation where powder IVM (Sigma-Aldrich) was mixed into all-purpose flour at 5% and then added to the cracked corn mixture to aid in even powder distribution. Chickens were fed *ad libitum* and feed consumed by each group was measured daily. Chickens were weighed daily and observed for clinical signs of toxicity, including diarrhea, mydriasis, ptosis, stupor and ataxia. The amount of chicken feed consumed was compared between groups using the students t-test and chicken growth rates were compared using linear regression. Blood was collected from these chickens through venipuncture at the end of their IVM diet regimen and for two days following IVM diet withdrawal. Serum was then isolated from the blood samples and stored at -80°C until further analysis.

Eurasian collared doves *(Streptopelia decaocto)* were captured by mist net in Wellington, CO and brought back to CSU. They were housed in groups of three and provided *ad libitum* clean water and either control diet or powder IVM formulation diet of 200 mg IVM/kg of diet for 10 days. Three doves were fed each control and powder IVM formulation diet and then used for mosquito bioassays.

Mosquito bioassays following blood feeding on birds were conducted on the last day of the IVM diet regimen for each group and for two days following IVM diet removal. For direct blood feeding on birds, the downy breast feathers were trimmed, and the exposed bird breast was placed on top of the mosquito cage. The birds were gently restrained for 30 minutes while the mosquitoes blood fed through the mosquito cage organdy. Given the difficulties of direct mosquito blood feeding on live chickens, supplemental serum-replacement membrane blood feeds were also performed, where frozen chicken serum was used in reconstituted blood meals using red blood cells from defibrinated calf blood [54,55].

All research with animals was reviewed and conducted under authorization by the Colorado State University Institutional Animal Care and Use Committee, protocol 16-6552A. Colorado State University Animal Care and Use is Public Health Service (PHS) and Office for Laboratory Animal Welfare (OLAW) assured (#A3572-01), United States Department of Agriculture (USDA) registered (#84-R-0003), and Association for Assessment and Accreditation of Laboratory Animal Care (AAALAC) accredited (#000834).

### IVM extraction and derivatization

All chemicals used in derivatization were HPLC grade and purchased from Sigma-Aldrich. IVM was extracted from serum following methanol precipitation [56]. 400 μL of methanol was added to 100 μL serum and vortexed for 1.5 min. Methanol precipitation was carried out at -80˚C overnight. Samples were centrifuged for 30 min at 16,000 *x g*. Supernatants were transferred and evaporated to dryness using a Speedvac concentrator (Savant). The dry residue was dissolved in 20 μL acetonitrile. Samples were derivatized according to previously published literature [57].

### HPLC quantification

A Waters 700 autosampler system was used to quantify IVM by high-performance liquid chromatography (HPLC)-fluorescence. A mobile phase of acetonitrile/water (3:1, v/v) was pumped through a C8 column (Waters, XBridge BEH C8 XP, 130 Å, 2.5 μm, 2.1x100 mm) at a rate of 0.45 mL/min. Excitation and emission spectra were 365 and 470 nm, respectively. 10 μL of derivatized sample was injected by the autosampler.

Precision was quantified as coefficient of variation (%CV). This was calculated interday and intraday, evaluating drug-free chicken serum samples (n = 5) spiked with IVM at 25, 50, and 100 ng/mL. Instrument CV was 6.11%. Intraday CV ranged between 4.36 and 9.77%. Interday reproducibility was 15.39%. Retention time CV was 1.77%.

The method was linear across the range of concentrations tested in the standard curve (3.125–100 ng/mL). Linear regression curves containing fortified IVM serum samples with concentrations of 3.125, 6.25, 12.5, 25, 50, and 100 ng/mL had a R-square value of 0.9974. Limits of detection and quantification were 1.56 ng/mL and 3.125 ng/mL, respectively.

### Pilot field trial

For the 2017 pilot trial, field sites were located in urban and suburban areas in the City of Fort Collins (mainly in city open space areas and near water sources) that were weekly mosquito trapping sites used by the city for WNV surveillance efforts and have been maintained since 2006 (S1 Fig). Six field sites were chosen based on historical WNV surveillance data from all city trapping sites as those having the highest number of WNV-positive *Cx*. *tarsalis* pools since 2006, while excluding trap sites in neighborhoods that are regularly treated with adulticides or used as sentinel sites for the Colorado WNV surveillance system by the state department of health. The 6 chosen sites were all in east Fort Collins and were randomly placed into the treatment group (3 sites; mosquito traps surrounded by IVM-treated bird feed stations) or the control group (3 sites; mosquito traps surrounded by control un-treated bird feed stations). At each field site, an array of three bird feed stations was placed in an approximate triangular perimeter around the mosquito trap at a distance of 50 m (S1 Fig). IVM-treated bird feed was used at a concentration of 200 mg/kg of diet, and the diet was a mixture of white proso millet, cracked corn, and flour (47.5:47.5:5, v/v/v). IVM-treated bird feed was changed daily to account for any effects of IVM degradation due to exposure, which also allowed for daily monitoring of any obvious adverse effects of IVM in local fauna.

Motion-activated trail cameras were used to document bird visits to feeders, with each field site having a motion-activated trail camera placed at one of the three feeders. Photos were screened using the Sibley Guide to Birds [58]. Due to an overabundance of pictures, only a random sampling of 6 days from the 2017 season were counted. Bird trapping and sampling of their blood was performed at two IVM sites. Birds were caught using mist nets placed approximately 10 m from an IVM-treated bird feeder. Blood was collected from netted birds using jugular venipuncture and placed into serum separator tubes. Bird sera were analyzed using HPLC-fluorescence and a subset of samples was analyzed using LC-MS. Because 200 μL of blood could not be drawn from the sparrows as needed for HPLC-fluorescence quantification, IVM analysis for sparrows was only documented as presence or absence. Control sera from house sparrows caught in spring 2014 were used as negative controls. Serum from one IVM-positive grackle was also used in a serum-replacement blood feed with colony *Cx*. *tarsalis* for a mosquito survival bioassay.

Mosquitoes were processed as part of the Fort Collins WNV surveillance program according to established protocols [59]. Briefly, mosquitoes were collected weekly by Vector Disease Control International using miniature CDC light traps baited with CO_2_. Mosquitoes were sorted to species and pooled into groups of typically no more than 50. Mosquito pools were screened at CSU using qRT-PCR using the following primer sequences: forward 5’ 1160-TCAGCGATCTCTCCACCAAAG 3’, reverse 5’ 1209-GGGTCAGCACGTTTGTCATTG 3’, probe 5’ FAM-1186-TGCCCGACCATGGGAGAAGCTC 3’ [59].

Bird sampling was done under Colorado Parks and Wildlife Scientific Collection License #17TRb2104 and Fort Collins Natural Areas Permit #914–2017.

### Statistical analysis

Chicken feed consumption was compared between groups using a t-test. Linear regression was done on chicken weights and the rate of weight gain was compared using Analysis of Covariance.

For mosquito bioassays, survival was analyzed using Kaplan-Meier survival curves and compared using Mantel-Cox (log-rank) test. LC_50_ values were calculated using a nonlinear mixed model with probit analysis [53]. IVM sera concentrations from chickens were compared using ANOVA. IVM sera concentrations from individual chickens were correlated to cumulative mosquito morality from bioassays conducted on the respective chickens using Spearman correlation.

The field trial utilized control and treatment sites located in the City of Fort Collins; however, it was an exploratory trial to test a new trial design and sites, and so was not powered for detecting differences in *Cx*. *tarsalis* abundance and WNV infection. *Cx*. *tarsalis* abundance from control and treatment sites were compared against each other using a generalized linear mixed model with negative binomial distribution that included site, week of trapping, and treatment. *Cx*. *tarsalis* abundance was also shown in comparison to historical data from 2006–2016 (which lacked any bird feed stations surrounding the traps). WNV infection rate was calculated as maximum likelihood estimate (MLE) using the Excel PooledInfRate Add In [60], but Fisher’s exact test was again used to compare the total number of WNV-positive and WNV-negative pools between control and treatment sites.

Statistical analyses were done in GraphPad Prism (Version 7) and R (Version 3.3.1).

## Results

### IVM has mosquitocidal effects in *Cx*. *tarsalis*

Mosquitocidal concentrations of IVM, selamectin, and eprinomectin were determined with mosquito bioassays following blood feeds with serially diluted drug (S2 Fig). IVM had the lowest LC_50_ concentration at 49.94 ng/mL (Table 1) as compared to eprinomectin with a LC_50_ of 101.59 ng/mL and selamectin with a LC_50_ of 151.46 ng/mL. With the lowest effective concentrations, ivermectin was chosen for further characterization in birds.

**Table 1:** Lethal concentrations (LC_x_) of ivermectin (IVM) for *Cx*. *tarsalis* when added to membrane blood meals.

LC_(x)_	[IVM] (ng/mL)
LC_5_	19.35 [10.52–26.80]
LC_10_	23.86 [14.29–31.62]
LC_25_	33.85 [23.58–42.10]
LC_50_	49.94 [39.71–59.93]
LC_75_	73.66 [61.37–92.96]
LC_100_	104.52 [84.38–149.54]

Brackets indicate 95% Confidence Intervals

**Fig 1:**
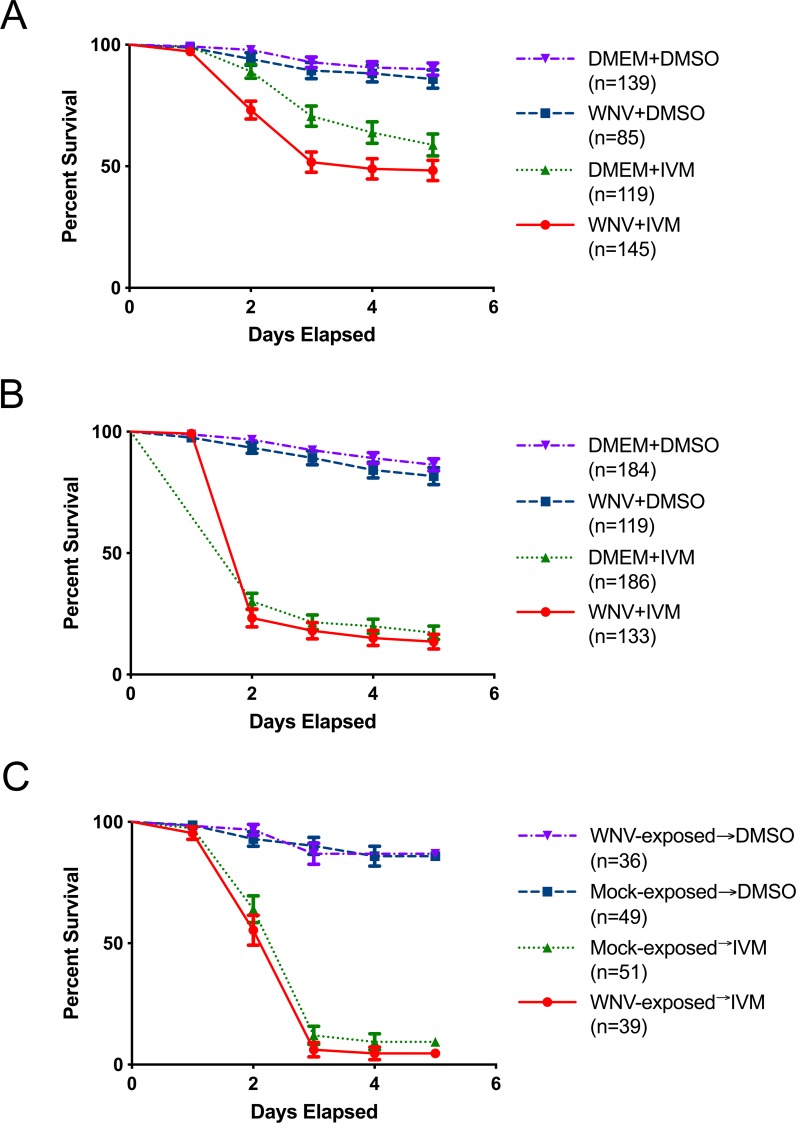
*Cx*. *tarsalis* survival in bioassays with IVM and WNV. (A) *Cx*. *tarsalis* survival following a membrane blood meal containing IVM at 73.66 ng/mL (LC_75_) and WNV at a titer of (A) 5x10^5^ PFU/mL or (B) 10^7^ PFU/mL. (C) *Cx*. *tarsalis* survival following a second membrane blood meal of IVM at 73.66 ng/mL given 10 days after a first blood meal of 10^7^ PFU/mL WNV. Error bars indicate standard error.

Potential interactions of IVM and WNV on *Cx*. *tarsalis* mortality were assessed in a simultaneous blood meal containing IVM (LC_75_) and WNV. Feeding with IVM only resulted in significantly increased mortality compared to DMSO controls; however, the observed 41% and 83% mortality for IVM control groups (Fig 1A and 1B) reflect the variability of mosquito bioassays, especially for intermediate ranges of lethal concentrations. WNV (both low and high titer) exposure in the absence of IVM did not affect *Cx*. *tarsalis* mortality over 5 days immediately after the blood meal (Fig 1A and Fig 1B), or following a second untreated blood meal 10 days later (Fig 1C). On the other hand, *Cx*. *tarsalis* given a concurrent blood meal containing low-titer WNV and IVM exhibited significantly increased mortality at 51% compared to the control IVM group not fed WNV with 41% morality (p = 0.0268, χ^2^ = 4.904) (Fig 1A). However, there was no significant difference (p = 0.2529, χ^2^ = 1.307) in mortality between *Cx*. *tarsalis* fed a concurrent blood meal containing high titer WNV and IVM compared to the control (Fig 1B). Similarly, *Cx*. *tarsalis* given a first blood meal of either DMEM control or high titer WNV, and then a second blood meal containing IVM 10 days later, showed no significant differences in mortality (p = 0.1637, χ^2^ = 1.940) (Fig 1C).

### Birds safely consume IVM-treated diet

Over 7 days of observation, there were no observable clinical signs of IVM neurotoxicity—diarrhea, mydriasis, ptosis, stupor, and ataxia–in groups that consumed either liquid Ivomec or powder formulations of IVM of 200 mg IVM/kg of diet.

For the Ivomec formulation diet, the chickens consumed an average 59.3 g of feed per chicken daily. This was significantly less than the corresponding control group which averaged 121.6 g of feed per chicken per day (p = 0.0045, t = 3.490). Consequently, there was also a significant difference (p <0.0001, F = 19.45) in the rate of weight gain between Ivomec and control groups (S3A Fig). For the powder IVM formulation diet, the IVM group consumed 60.97 g of feed per chicken each day, which was not significantly different from daily control group consumption of 55.2 g of feed per chicken (p = 0.2928, t = 1.100). This was also reflected in similar rates of weight gain between powder IVM and control groups (p = 0.0680, F = 4.022) (S3B Fig).

### Blood feeding on IVM-treated birds resulted in significantly increased *Cx*. *tarsalis* mortality

*Cx*. *tarsalis* mortality following blood feeding on IVM-treated chickens increased as IVM concentration within the diet increased (S4 Fig). There were significant differences in mosquito mortality following blood feeding on chickens given 50 mg IVM/kg of diet (p = 0.0132, χ^2^ = 6.146) and 100 mg IVM/kg of diet (<0.0001, χ^2^ = 86.48). However, the largest increase in mortality (p<0.0001, χ^2^ = 461.1) following blood feeding was at 200 mg IVM/kg of diet with 95.2% mortality in mosquitoes fed on IVM-treated chickens and 2.7% mortality in mosquitoes fed on control chickens. All subsequent experiments used IVM-treated feed at 200 mg IVM/kg of diet.

**Fig 2:**
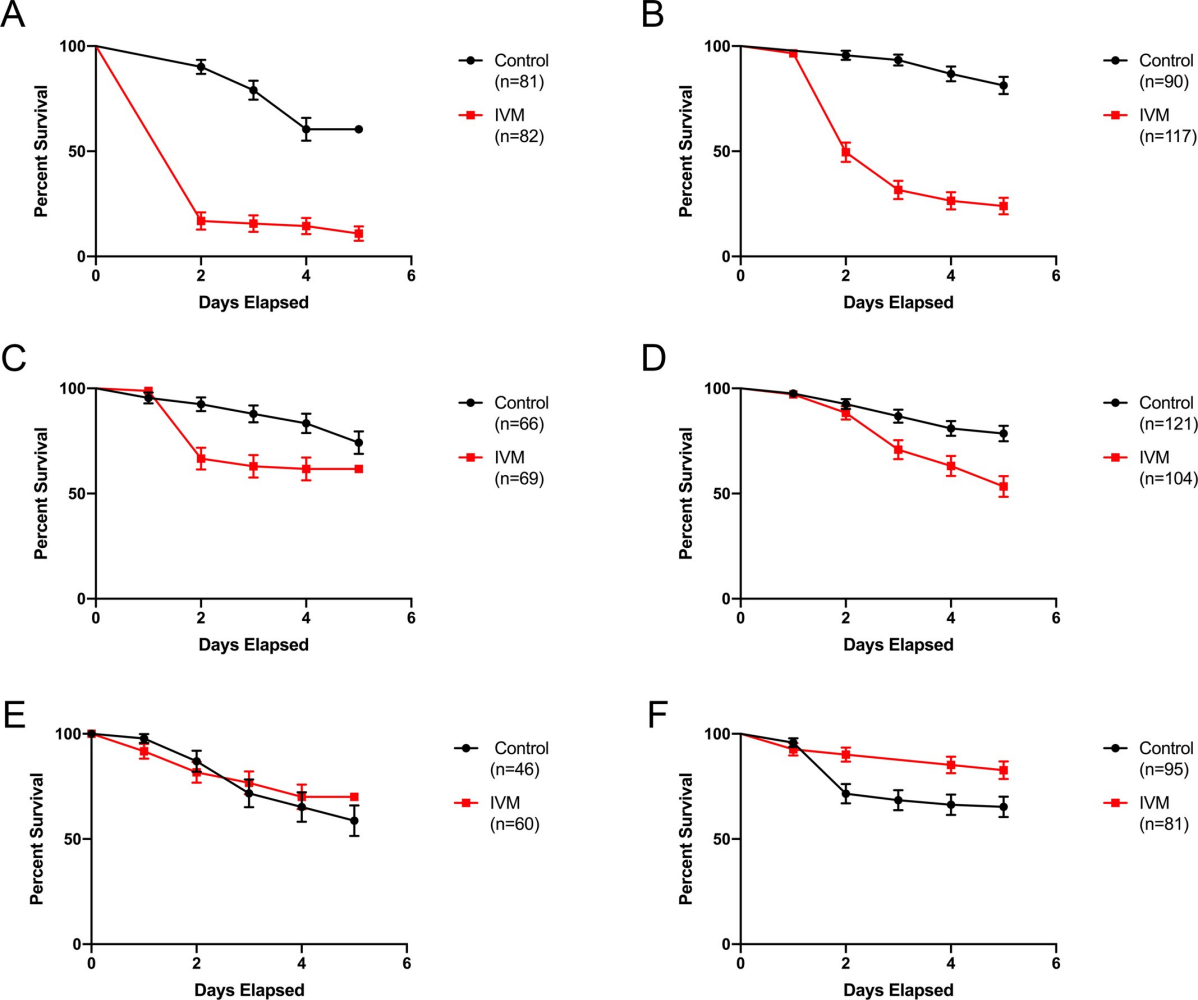
Blood feeding on Ivomec-treated chickens increased *Cx*. *tarsalis* mortality. *Cx*. *tarsalis* survivorship following direct blood feeding on chickens given Ivomec-formulation diet at a concentration of 200 mg IVM/kg of diet for 3 (left panels: A, C, E) and 7 (right panels: B, D, F) days. (Top panels: A, B) Blood feeding occurred on the last day treated diet was given to the IVM groups. (Middle panels: C, D) Blood feeding occurred one day after treated diet was withdrawn from the IVM groups. (Bottom panels: E, F) Blood feeding occurred on the second day after treated diet was withdrawn from the IVM groups. Error bars indicate standard error.

For the Ivomec formulation at 200 mg IVM/kg of diet, there was a significantly increased mortality in mosquitoes blood fed on chickens consuming Ivomec-diet for either 3 or 7 days as compared to mosquitoes blood fed on control chickens (Fig 2; left and right panels, respectively). On the last day of Ivomec feed administered, for both 3 or 7 days, there was a significant increase (p<0.0001, χ^2^ = 80.22 and χ^2^ = 76.41, respectively) in mortality between mosquitoes blood fed on chickens consuming an Ivomec diet with upwards of 80% mortality as compared to mosquitoes blood fed on control chickens with less than 40% mortality (Fig 2A and 2B). This difference in mosquito mortality between treatment and controls decreased when the blood feed occurred 1 day following the withdrawal of the Ivomec diet in the treatment group (Fig 2C and 2D). After 2 days following Ivomec diet withdrawal, there was no significant difference in mosquito mortality between those blood fed on Ivomec-consuming chickens as compared to mosquitoes blood fed on control chickens in the 3 day group, but there was a significant difference in the 7 day IVM group (p = 0.0117, χ^2^ = 6.354) which is likely due to the variability in mosquito bioassays (Fig 2E and 2F). In addition, the time administered Ivomec-treated diets (3 vs. 7 days) did not affect mosquito survival curves following direct blood feeding on chickens, regardless if the mosquitoes were blood fed on the last day of chicken time on the diets, or if the chickens were 1 or 2 days post withdrawal of the diets (Fig 2, left vs. right panels).

There was also significantly increased mosquito mortality in mosquitoes blood fed on chickens consuming the powder formulation of IVM (200 mg IVM/kg of diet) compared to mosquitoes fed on control chickens (S5 Fig). Because bioassays from the Ivomec formulation and a preliminary powder formulation indicated no differences between mosquitocidal effects for groups given IVM for 3 or 7 days, these and subsequent experiments focused on the 7 day time point. A direct blood feed of mosquitoes on chickens given a powder IVM diet for 7 days resulted in 92.3% mosquito mortality as compared to 25.7% mosquito mortality from those blood fed on control chickens (p<0.0001, χ^2^ = 41.23) (S5A Fig), while an indirect, serum-replacement blood feed using sera from chickens given a powder IVM diet for 7 days resulted in 79.0% mosquito mortality as compared to 16.7% mortality from those blood fed on control chicken serum (p<0.0001, χ^2^ = 42.83) (S5B Fig). Furthermore, the mosquito survival curves between those blood fed directly on IVM-treated chickens as compared to sera from IVM-treated chickens were significantly different (red lines in S5A Fig vs. S5B; p<0.0001; hazard ratio 2.007). At 1 day post-powder IVM diet withdrawal, there was still a significant difference (p = 0.001, χ^2^ = 10.86) in mosquito mortality between those directly blood fed on IVM-diet vs control-diet chickens (S5C Fig; 90.9% vs. 0% mortality). However, this mosquitocidal effect was not apparent in a serum-replacement blood feed derived from chicken blood taken 1 day after IVM diet withdrawal (p = 0.7445, χ^2^ = 0.1062) (S5D Fig). As above, the mosquito survival curves between those blood fed directly vs. indirectly on treated chickens 1 day post-diet withdrawal were also significantly different (red lines in S5C Fig vs. S5D; p<0.0001; hazard ratio 6.742). At 2 days post-IVM diet withdrawal, blood/serum from treated chickens was no longer mosquitocidal in either direct blood feeding (p = 0.8402, χ^2^ = 0.04065) or serum-replacement (p = 0.1792, χ^2^ = 1.804) assays (S5E and S5F Fig).

**Fig 3:**
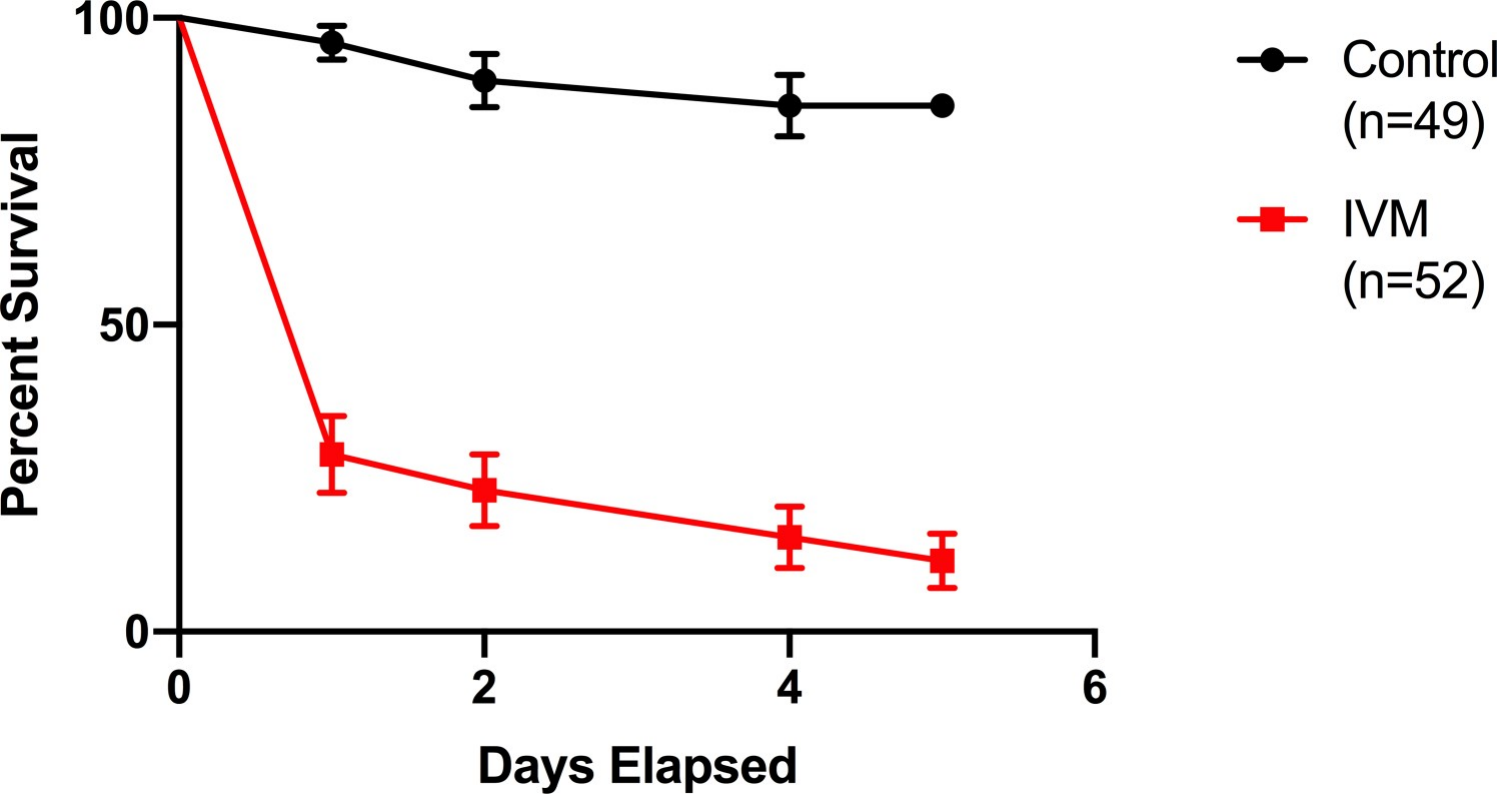
Blood feeding on powder IVM-treated doves increased *Cx*. *tarsalis* mortality. *Cx*. *tarsalis* survival following direct blood feeding on captured wild Eurasian collared doves fed powder IVM-diet at a concentration of 200 mg IVM/kg diet for 7 to 10 days. Error bars indicate standard error.

Direct blood feeds of *Cx*. *tarsalis* were also conducted on six wild caught Eurasian Collared Doves fed either a powder IVM formulation diet of 200 mg IVM/kg or control diet in the laboratory (Fig 3). There was a significant difference in mosquito mortality (p<0.0001, χ^2^ = 60.34) with 88.5% mortality in *Cx*. *tarsalis* fed on IVM-treated doves as compared to 14.3% mortality from mosquitoes blood fed on control doves. Additionally, there were no clinical signs of IVM toxicity observed in this treated bird species.

### Chicken IVM serum concentrations correlated with *Cx*. *tarsalis* mortality in bioassays

**Fig 4:**
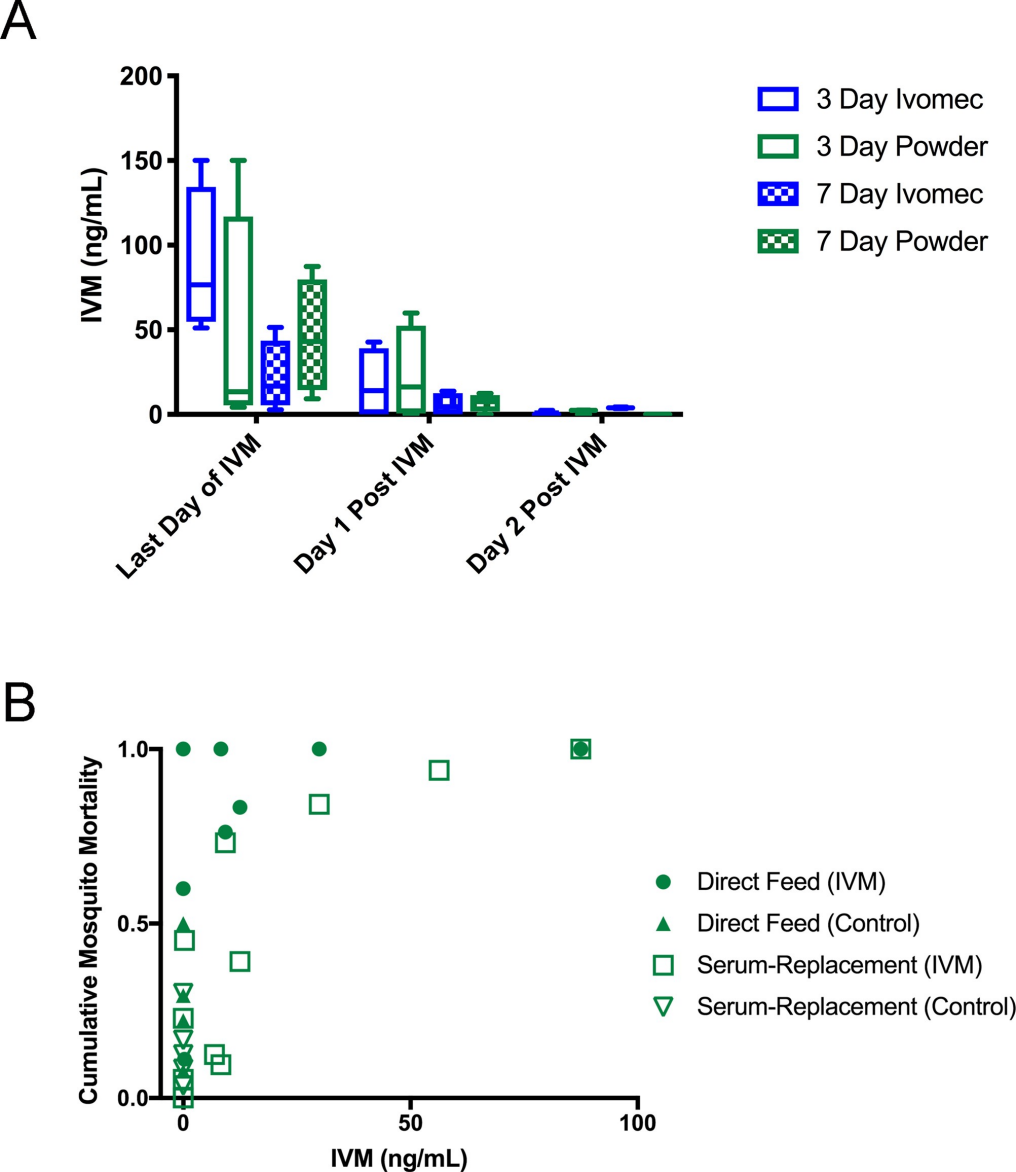
Characterization of chicken IVM sera concentrations. (A) Chicken sera IVM concentrations measured in IVM-treated groups and taken on the last day treated diet was given, or one or two days after treated diet was withdrawn. Lines indicate median values, boxes indicate 25–75 percentiles, whiskers indicate minimum and maximum values. (B) Individual chicken IVM serum concentrations versus corresponding cumulative *Cx*. *tarsalis* mortality on day 5 post blood feeding for both direct and serum-replacement blood feeds on chickens given powder IVM-formulation feed at a concentration of 200 mg IVM/kg of feed.

Neither the IVM formulation nor the time for which the chickens consumed IVM-treated diet resulted in significant differences in average IVM serum concentrations (p = 0.2715, F = 1.472) (Fig 4A, blue vs. green bars). On the last day of IVM diet, the average IVM serum concentrations (with SD) were 88.575 (±43.613) ng/mL for 3-day Ivomec, 45.255 (±70.051) ng/mL for 3-day powder IVM, 21.910 (±20.914) ng/mL for 7-day Ivomec, 45.745 (±33.852) ng/mL for 7-day powder IVM. Chicken IVM serum concentrations decreased following withdrawal of the IVM diet and were nearly undetectable at 2 days post-withdrawal, which corresponded with mosquito bioassay results showing decreases in mosquitocidal activity following IVM-diet removal. Additionally, IVM serum concentrations were correlated to resulting mosquito mortality from blood feeding on these corresponding IVM-powder fed chickens (Fig 4B). There was a higher correlation between IVM serum concentrations and mortality from serum-replacement feeds with a Spearman r of 0.8629 (P = 0.0007), while the correlation between IVM serum concentrations and mortality from direct blood feeds was 0.4153 (p = 0.3062).

### Pilot field trial

For a pilot trial testing IVM feed in a natural transmission cycle, feeder stations were placed in urban and suburban areas within the City of Fort Collins (S1 Fig) and randomized to treatment or control sites. Bird visits to IVM feeders at all sites were dominated by grackles with infrequent visits by house (*Passer domesticus)* and sagebrush sparrows (*Artemisiospiza nevadensis)* and black-capped chickadees (*Poecile atricapillus)* (Table 2). There were also two visits by blue jays *(Cyanocitta cristata)*, and a few other birds which could not be identified from the photographs. A more homogenous mix of grackles, house and brewers *(Spizella breweri)* sparrows, blue jays, black-capped chickadees, bushtits, and squirrels visited control feeders (Table 2).

**Table 2:** 2017 bird visitation data as documented by field cameras on bird feeders.

Animal	Control Sites	IVM Sites
Grackle	92.5% (n = 1219)	22.8% (n = 31)
Blue Jay	0.15% (n = 2)	22.8% (n = 31)
Sagebrush sparrow	0.075% (n = 2)	1.5% (n = 2)
Squirrel	1.3% (n = 15)	19.9% (n = 27)
Raccoons	0.5% (n = 7)	
House sparrow	0.075% (n = 1)	0.74% (n = 1)
Black-capped chickadee	2.5% (n = 33)	6.6% (n = 9)
Unidentified	2.8% (n = 37)	3.7% (n = 5)
Bushtit		2.9% (n = 4)
Brewer’s sparrow		19.1% (n = 26)

Percentages indicate proportion of visits composed of indicated species and n indicates number of documented visits made by the indicated species.

**Fig 5:**
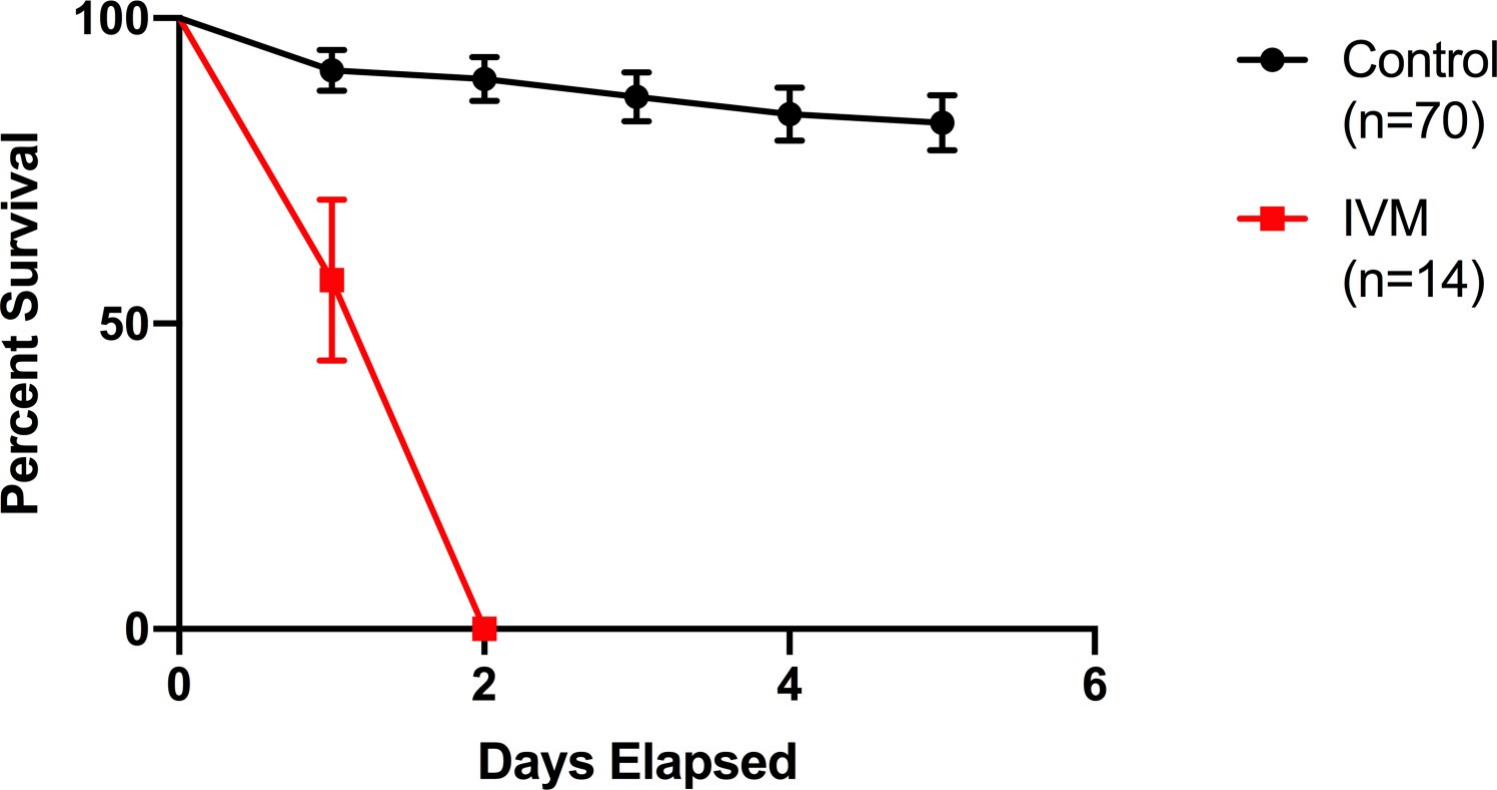
Blood feeding on wild-caught grackle serum resulted in increased *Cx*. *tarsalis* mortality. *Cx*. *tarsalis* survival following a serum-replacement blood feed using serum from a wild-caught grackle in which IVM was detected compared to control calf blood.

Birds were also caught by mist net and their sera assayed for IVM at the end of the field season. Ten grackles and 5 sparrows were caught over 4 mornings of sampling on August 30^th^ and September 2^nd^, 3^rd^, and 7^th^. Most birds had been observed feeding from the IVM-treated feeder immediately preceding mist net capture. Nine grackles and 4 sparrows (87% of tested sera) had detectable levels of IVM within their serum, and the negative control sparrow serum from 2014 had no detectable IVM (Table 3). Serum from grackle #5 (Table 3) was plentiful and thus further used in a LC-MS assay to confirm the presence of IVM, and also tested in a serum-replacement bioassay. Interestingly, even though the IVM serum concentration in grackle #5 was measured as 5.7 ng/mL, there was strong mosquitocidal effect from this serum (100% mortality within 2 days; p<0.0001, χ^2^ = 54.15) compared to control mosquitoes fed on control calf serum (Fig 5).

**Table 3:** Detection and quantification of IVM in field-caught birds in 2017.

Bird	IVM Detection	IVM Quantification
Sparrow 1	Positive	NA
Sparrow 2	Positive	NA
Sparrow 3	Positive	NA
Sparrow 4	Positive	NA
Sparrow 5	Positive	NA
Sparrow 6	Positive	NA
Sparrow 7	Positive	NA
Sparrow 8	Positive	NA
Sparrow 9	Positive	NA
Sparrow 10	Negative	NA
Grackle 1	Negative	NA
Grackle 2	Positive	Below LOQ
Grackle 3	Positive	Below LOQ
Grackle 4	Positive	Below LOQ
Grackle 5	Positive	5.7 ng/mL

Sera from field-caught birds was assayed for IVM. Sparrow sera were only used for detection due to low volumes available. Limit of quantification (3.125 ng/mL) is indicated as LOQ.

**Fig 6:**
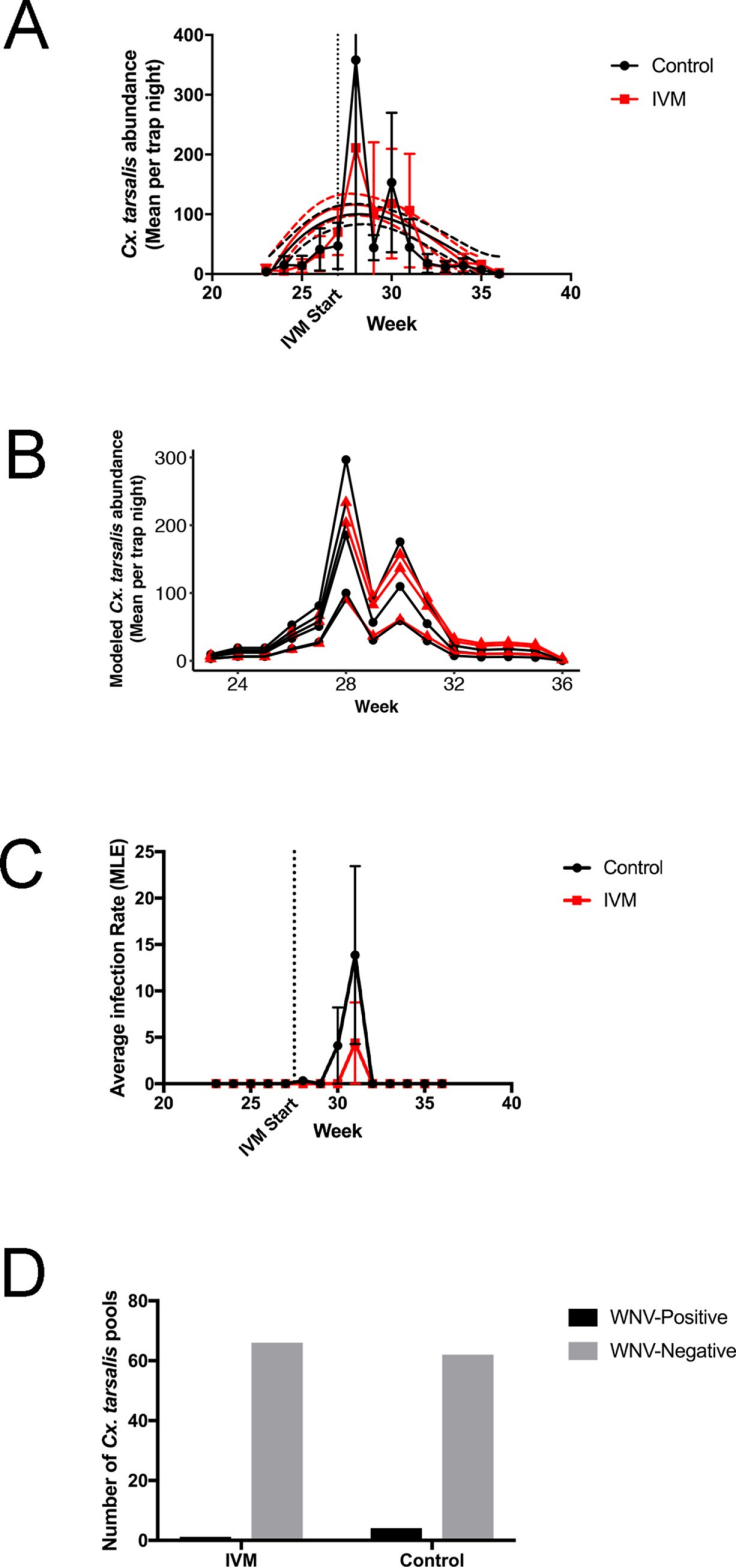
*Cx*. *tarsalis* abundance and infection for 2017 field sites. Dotted lines indicate the start of IVM-treated feed at IVM sites. (A) Historical data from the same sites (2006–2016) were fit to non-linear regression (smoothed solid lines) with 95% CIs (smoothed dashed lines), and 2017 data were plotted as connected points. (B) Modeled *Cx*. *tarsalis* abundance from each IVM (red) and control (black) site over the season. Red lines indicate IVM treatment. (C) Average infection rate (MLE) from IVM and control sites. (D) Number of WNV-positive and WNV-negative pools at IVM and control sites is shown.

*Cx*. *tarsalis* abundance over time in 2017 at the urban and suburban field sites was similar to historical data collected from the same traps for 10 years prior (Fig 6A). A generalized linear mixed model with negative binomial distribution did not find a significant difference between *Cx*. *tarsalis* abundance at IVM sites compared to control sites (p = 0.161, z = 1.401) (Fig 6B). The low number of WNV infections did not allow for robust statistical analysis, although MLE was calculated (Fig 6C). A combined Fisher’s Exact Test of all 6 field sites showed a non-significant decrease in the proportion of WNV-positive pools to WNV-negative pools among control and treatment traps (p = 0.2081) (Fig 6D).

## Discussion

This study presents a novel characterization of IVM-treated bird feed as a systemic endectocide to control WNV transmission. Lab studies characterized the effects of IVM-treated bird feed in both domestic and wild birds, especially mosquitocidal effects in *Cx*. *tarsalis* blood fed on birds consuming this IVM-containing diet. In addition, a pilot field trial was performed over a WNV season to gather preliminary efficacy data on the effects of IVM-treated bird feed within a natural WNV transmission cycle between wild birds and mosquitoes.

IVM was determined to be the most effective endectocide tested with the lowest lethal concentrations for *Cx*. *tarsalis*. In addition, there did not appear to be a synergistic effect of IVM and WNV on *Cx*. *tarsalis* mortality in either a simultaneous blood feed of IVM and high titer WNV or sequential blood feeds, the first containing WNV and the second containing IVM. There was a statistical difference between survival curves of *Cx*. *tarsalis* fed a concurrent blood meal of a low WNV titer IVM compared to *Cx*. *tarsalis* fed only IVM. However, this increased mortality was likely due to the variable survival response of mosquitoes to IVM particularly at intermediate lethal concentrations, rather than a biologically significant interaction between WNV and IVM as there was no mortality difference between mosquitoes fed a concurrent higher titer WNV+IVM blood meal compared to mosquitoes fed DMEM+IVM. There was also no difference between mosquitoes previously exposed to WNV and then fed IVM as compared to mosquitoes unexposed to WNV and then fed IVM. While there is a study suggesting that IVM can inhibit WNV replication by targeting NS3 helicase activity, this was an *in vitro* cell-culture study using mammalian cells, and the concentration of IVM needed to inhibit 50% of the RNA synthesis in the Vero cells infected with WNV was considerably higher than what was achieved in our chickens following IVM feed consumption [61].

No clinical signs of toxicity were observed in any of the birds consuming either formulation of IVM feed. This was not surprising as IVM is given therapeutically in bird species in a wide range of doses (0.2 mg/kg to 2 mg/kg), depending on route of administration. However, more detailed studies of IVM toxicity should be conducted in multiple bird species in future controlled experiments. Previous studies have identified neurotoxic effects in pigeons following long-term consumption of a diet containing avermectin [62,63], of which IVM is a safer derivative [64]. Specifically, Chen et al. observed clinical signs of neurotoxicity, ranging from reduced activity and food intake following avermectin consumption for 60 days on a 20 mg/kg diet, to ataxia and spasms following avermectin consumption for 30 days on a 60 mg/kg diet [63]. On the other hand, a characterization of IVM pharmacokinetics, metabolism, and tissue distribution in laying hens treated intravenously (400 μg/kg) or consuming IVM-treated water (400 μg/kg/day) for 5 days did not report any ill effects in the birds [51]. Following the intravenous injection of the hens, the highest IVM plasma concentrations (739.6 ± 50.2 ng/mL) were 30 minutes after administration and plasma concentrations remained below 10 ng/mL after 24 hours [51]. Mean IVM concentrations in our chickens fed exclusively on an IVM-containing diet for 3 and 7 days were approximately 45 ng/mL, and similarly we did not observe any neurotoxicity. It remains to be determined if these results vary among different bird species or longer times on the diet. However, in the field studies, it is unlikely that the IVM-treated bird feed was the sole or even primary source of food for the wild birds visiting the feeders given the abundance of alternative food sources during summer.

While chickens on the powder IVM and control diets consumed equivalent quantities of food, there was a significant difference in feed consumption among chicken fed the Ivomec diet and their controls. This may be a result of the glycerol formal and propylene glycol carriers in Ivomec that could give an unpleasant taste, as propylene glycol has been identified as a unpleasant and unpalatable feed additive in cattle [65]. Consequently, the decreased Ivomec feed consumption relative to control feed consumption is likely responsible for the significantly reduced rate of weight gain in the Ivomec group as compared to controls.

Chickens that consumed either a powder IVM or Ivomec diet reached mosquitocidal levels of IVM in their blood within 3 days, as demonstrated by both the IVM serum concentrations in the chickens as well as the significant difference in survival curves of mosquitoes blood fed on IVM-treated chickens compared to controls. There were no notable differences between either IVM diet formulations in mosquitocidal efficacy when considering either time to achieve a mosquitocidal effect and IVM persistence in chicken serum following IVM withdrawal. Furthermore, the time the chickens were placed on the two IVM diets (3 and 7 days) did not significantly affect mosquito mortality, serum concentrations, or the elimination time of IVM from serum following feed withdrawal. This is corroborated by the similar IVM serum concentrations at all time points among the different IVM administration times and formulations. A mosquitocidal effect, but no observable bird toxicity, was demonstrated for wild-caught Eurasian collared doves following consumption of the 200 mg IVM/kg diet, indicating similar mosquitocidal efficacy of the approach in one other bird species and thus potential application to other wild bird species in field settings.

The mosquito mortality in control groups had a greater variation for direct blood feeds (17.75% CV) relative to control groups for serum-replacement blood feeds (3.57% CV), indicating that direct blood feeds results in more inherent variability in mosquito mortality. This increased variability could be a result of increased mosquito handling and rougher conditions during direct blood feeding on birds. It is also possible this higher variability is partly due to smaller sample sizes from the direct blood feeds due to the low success of our colony mosquitoes imbibing full blood meals from live chickens. Regardless, the higher variability among direct blood feed data led to a weaker correlation between IVM serum concentrations and mosquito mortality compared to that from serum-replacement blood feed data. However, despite this higher variability, cumulative mosquito mortality from these direct blood feeds was higher (consistently above 75%) compared to that from the serum-replacement feeds, and mostly independent of measured IVM concentration in the chickens’ sera. One likely possibility for this discrepancy is that the IVM concentration within serum extracted from venous blood may not always be an accurate representation of the IVM concentration in subdermal capillary blood on which mosquitoes blood feed. It has been previously proposed that because IVM is extremely lipophilic and sequestered in fatty tissues, there may exist a concentration gradient of higher IVM or IVM metabolite concentrations in adipose tissue and blood of the surrounding capillaries compared with venous blood [66]. This is also one explanation for the observation that the IVM serum concentrations in chickens correlated with higher cumulative mosquito mortality than would be predicted from the LC_x_ values calculated using artificial membrane feeds. A useful future analysis would be to compare mosquito mortality results from direct skin blood feeding on chickens, membrane blood feeds using venous blood drawn from the chickens, and serum replacement blood feeds using unfrozen serum from the same chickens.

The mosquitocidal effect from chickens on an IVM-containing diet did not extend past one day after IVM-feed withdrawal, and this corresponded with the IVM serum concentrations that were generally below detectable limits by two days post-IVM feed withdrawal. This could potentially be a concern for applying this strategy in the field as it would suggest that frequent bird visits would be necessary to maintain their mosquitocidal blood concentrations of IVM. However, our field data indicated that wild birds were visiting the bird feeders and did have detectable levels of IVM within their sera during multiple days throughout the trial. In addition, one grackle from our 2017 field trial had strongly mosquitocidal serum as assessed in a bioassay, even though the IVM concentration in that serum was surprisingly low. It is promising that a majority of the birds tested had detectable levels of IVM within their sera, indicating that there was an unexpectedly high coverage of IVM in captured birds. However, the placement of mist nets at roughly a 10 m distance from an IVM feeder may have biased the sampling towards birds that visited the feeder, so future studies should more intensively sample birds at wider radii from the feeders. Understanding IVM coverage and persistence within wild birds is an important component of determining the efficacy of this strategy and should be supplemented with detection of IVM in wild-caught blood fed *Cx*. *tarsalis* in future field seasons. This could also be coupled with mosquito survival bioassays using wild bird sera to assess mosquitocidal activity as we performed here.

This use of IVM-treated feed as a systemic endectocide to control WNV transmission is based on targeting *Cx*. *tarsalis* by medicating its preferred host species. Previous studies in California implicate *Cx*. *tarsalis* as a regionally adaptive, opportunistic blood feeder with a preference for avian hosts, and the diversity of available blood meal sources is reflected in the composition of its blood meals [67–71]. Important avian hosts for *Cx*. *tarsalis* in small rural towns within Weld County, which is adjacent to our Fort Collins field site area, include American Robins, doves, and other Passeriformes [33]. American Robins are an important *Cx*. *tarsalis* blood meal source and WNV amplification host that does not frequent bird feeders and would not be targeted by this current strategy [33,72,73]. However, doves and passerines are preferred blood meal sources of *Cx*. *tarsalis* and contribute to the cumulative number of WNV-positive *Cx*. *tarsalis* at estimated rates of approximately 30% in June, 60% in July, and 85% in August [33]. This represents a large proportion of *Cx*. *tarsalis* blood meal sources and WNV-positive contributions from birds that consume grain and seed that could be targeted throughout the summer season. However, our trail camera data did not show a large proportion of visits from these species identified as regionally important. For example, grackles were predominantly visiting our IVM-treated feeders, while control feeders were visited mostly by grackles, blue jays, brewer’s sparrows, and squirrels. However, the single trail camera we employed per site may not have fully documented bird visits to other feeders at the field site. Camera placement was limited to tree-filled areas where a feeder could be placed with a camera locked to a tree across from the feeder, and this may have biased the camera data against bird species that feed in open space or brush rather than among trees. This limitation of the field camera data is illustrated by our detection of IVM in house sparrows caught by mist net, but we had no documentation of sparrow visits on the trail camera for this specific field site. An important future direction will also be to gather a more updated understanding of the *Cx*. *tarsalis* blood meal sources within urban and suburban area of the City of Fort Collins, which might allow for specific targeting of these bird species with attractive bird feed compositions and an optimized bird feeder design.

In addition to a better characterization of avian blood meal sources for *Cx*. *tarsalis*, a more complete understanding of bird and *Cx*. *tarsalis* spatial dynamics is also important for determining the best placement for the IVM-treated feeders. Because our field sites were chosen based on historical mosquito and WNV surveillance, we did not account for crucial bird parameters that may have influenced mosquito sampling. For example, birds may have fed at the IVM-treated feeders and returned to their communal roosts where they would have been blood fed on by *Cx*. *tarsalis* [33,70,74], representing a treatment effect in a different population of *Cx*. *tarsalis* than sampled at our traps. Accounting for these bird-mosquito spatial dynamics by placing IVM-treated feeders near communal roosts of granivorous birds and sampling mosquitoes within close range may show the greatest entomological treatment effect, especially as Kent et al. gives an example of a house sparrow roost serving as both a major blood meal and amplification source of WNV-positive *Cx*. *tarsalis* [33]. While communal bird roosts could present a critical target, this strategy should continue to be tested in areas of increased human use such as parks and backyards. This highlights that future studies should also consider the best placement of bird feeders in the context of both human land use, and bird and mosquito interactions.

Our pilot field trial was ultimately inconclusive and did not find a significant difference in *Cx*. *tarsalis* abundance or WNV infection due to IVM treatment. This is likely due to three field sites for each trial arm being underpowered to observe a significant effect. However, these preliminary field data will serve as important effect size variables with which to properly power future field trials. In addition, this strategy of controlling vector pathogen transmission with an endectocide like IVM is based on shifting the mosquito population age structure in a treatment area from older, infectious mosquitoes to younger, non-infectious mosquitoes, and is less dependent on reducing total mosquito abundance. This has been modeled, as well as observed with empirical data, in trials testing IVM for malaria transmission control [75,76]. We would also expect to see a shift in the age structure of the population to fewer older, infectious *Cx*. *tarsalis* and more uninfected, younger mosquitoes. However, our preliminary results from ovary dissections and parity scoring according to Detinova [77] showed consistently high parous rates within the field-caught *Cx*. *tarsalis*. This suggested that autogeny, or the ability to develop a batch of eggs without imbibing a blood meal, could be present among the *Cx*. *tarsalis* in our study area and confounded our data, and we chose to not conduct further parity scoring during our pilot field trial. As determining age structure of the wild *Cx*. *tarsalis* population would be additional way to evaluate this control strategy, future studies should integrate other age-grading techniques such as near infrared spectroscopy (NIRS) [78,79].

Our characterization of IVM as a systemic endectocide in birds demonstrates its feasibility to be developed into a novel WNV transmission control tool. We have demonstrated that birds readily consume IVM-treated feed in the lab and field with our formulation and concentration, while not displaying any observable clinical signs of toxicity following consumption. Furthermore, *Cx*. *tarsalis* mosquitoes blood feed on these IVM-treated birds and often die as a result. Our pilot field trial testing IVM-treated feed in natural transmission cycles within wild birds and mosquitoes was ultimately inconclusive, but did provide critical effect size variables to inform future trial design. Important future directions will be to optimize treated bird feed formulations for the field and better characterize the pharmacokinetics and pharmacodynamics of this diet within multiple bird species, especially in relation to mosquitocidal activity and physiological/clinical signs of toxicity. In addition, a more-updated, regionally-specific understanding of the blood meal host preferences of *Cx*. *tarsalis* across urban, suburban and rural habitats would allow for better targeting of these preferred host species through the design of an attractive bird feed composition, discriminating bird feeders, and optimized bird feeder location for application to different geographic areas. Finally, our field study provides an important template for future field studies across multiple WNV seasons that will be adequately-powered for measuring effect sizes in entomological and other outcomes.

## Supporting information

S1 Fig Schematic of 2017 pilot trial field sites. Panel A depicts the WNV surveillance trap sites within the city of Fort Collins. The 3 control (black circles) and 3 IVM sites (red circles) are shown. Panel B shows a representative field site with an array of 3 bird feeders (red squares) surrounding one mosquito trap (yellow circle). The figure was created using LandsatLookViewer (http://landsatlook.usgs.gov/).
(TIF)

S2 Fig ***Cx*. *tarsalis* mortality following blood feeding on IVM (A), selamectin (B), and eprinomectin (B).** *Cx*. *tarsalis* were blood fed on increasing concentrations of endectocides and their mortality was observed to calculate lethal concentrations. Error bars indicate standard error.
(TIF)

S3 Fig Average chicken weight gain over time. Linear relationship between chicken weight and days elapsed is shown where black lines indicate control groups and red lines indicate ivermectin-treated groups of chickens fed (A) Ivomec-formulation diet or (B) powder IVM-formulation diet. Error bars indicate standard deviation.
(TIF)

S4 Fig *Cx*. *tarsalis* mortality increases when blood fed on chickens fed increasing concentrations of IVM-treated diet. *Cx*. *tarsalis* survival following direct blood feeding on chickens that were fed Ivomec-formulation diet for 7 consecutive days at concentrations of 50 mg IVM/kg of diet, 100 mg IVM/kg of diet, and 200 mg IVM/kg of diet. Error bars indicate standard error.
(TIF)

S5 Fig Blood feeding on powder IVM-treated chickens increased *Cx*. *tarsalis* mortality. *Cx*. *tarsalis* survivorship following direct (left panels: A, C, E) or serum-replacement (right panels: B, D, F) blood feeding on chickens given powder-IVM diet at a concentration of 200 mg IVM/kg of diet for 7 days. (Top panels: A, B) Blood feeding occurred on, or using serum taken on, the last day treated diet was given to the IVM groups. (Middle panels: C, D) Blood feeding occurred on, or using serum taken on, the day after treated diet was withdrawn from the IVM groups. (Bottom panels: E, F) Blood feeding occurred on, or using serum taken on, the second day after treated diet was withdrawn from the IVM groups. Error bars indicate standard error.
(TIF)
