# Supplementary figures and images for: Evaluation of a novel West Nile virus transmission control strategy that targets Culex tarsalis with endectocide-containing blood meals
Source: PLoS Negl Trop Dis. 2019 Mar 7;13(3):e0007210. doi: 10.1371/journal.pntd.0007210 (PMC6424467; doi:10.1371/journal.pntd.0007210)

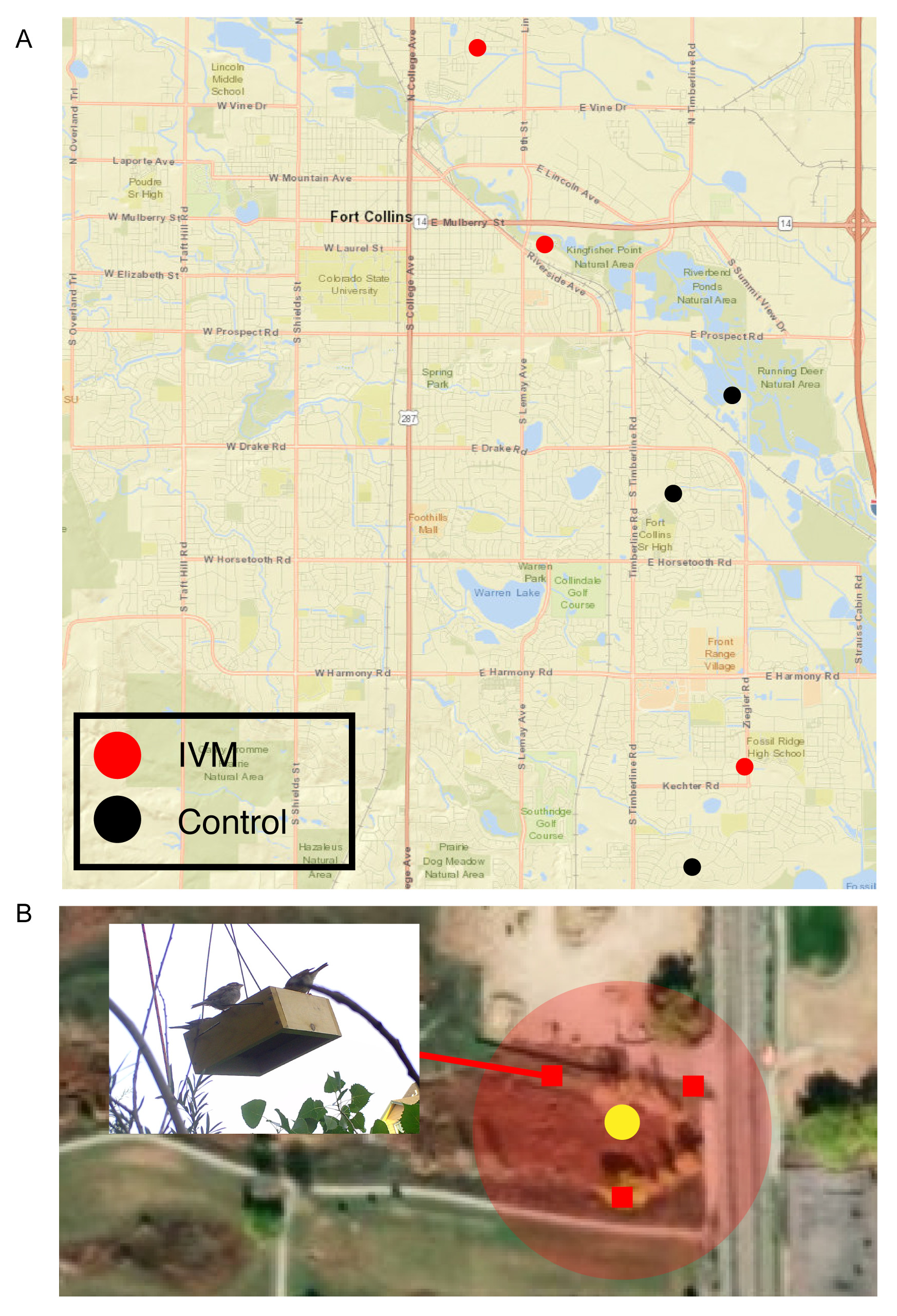

Supplement: S1 Fig — Panel A depicts the WNV surveillance trap sites within the city of Fort Collins. The 3 control (black circles) and 3 IVM sites (red circles) are shown. Panel B shows a representative field site with an array of 3 bird feeders (red squares) surrounding one mosquito trap (yellow circle). The figure was created using LandsatLookViewer (http://landsatlook.usgs.gov/). (TIF) [file pntd.0007210.s001.tif]

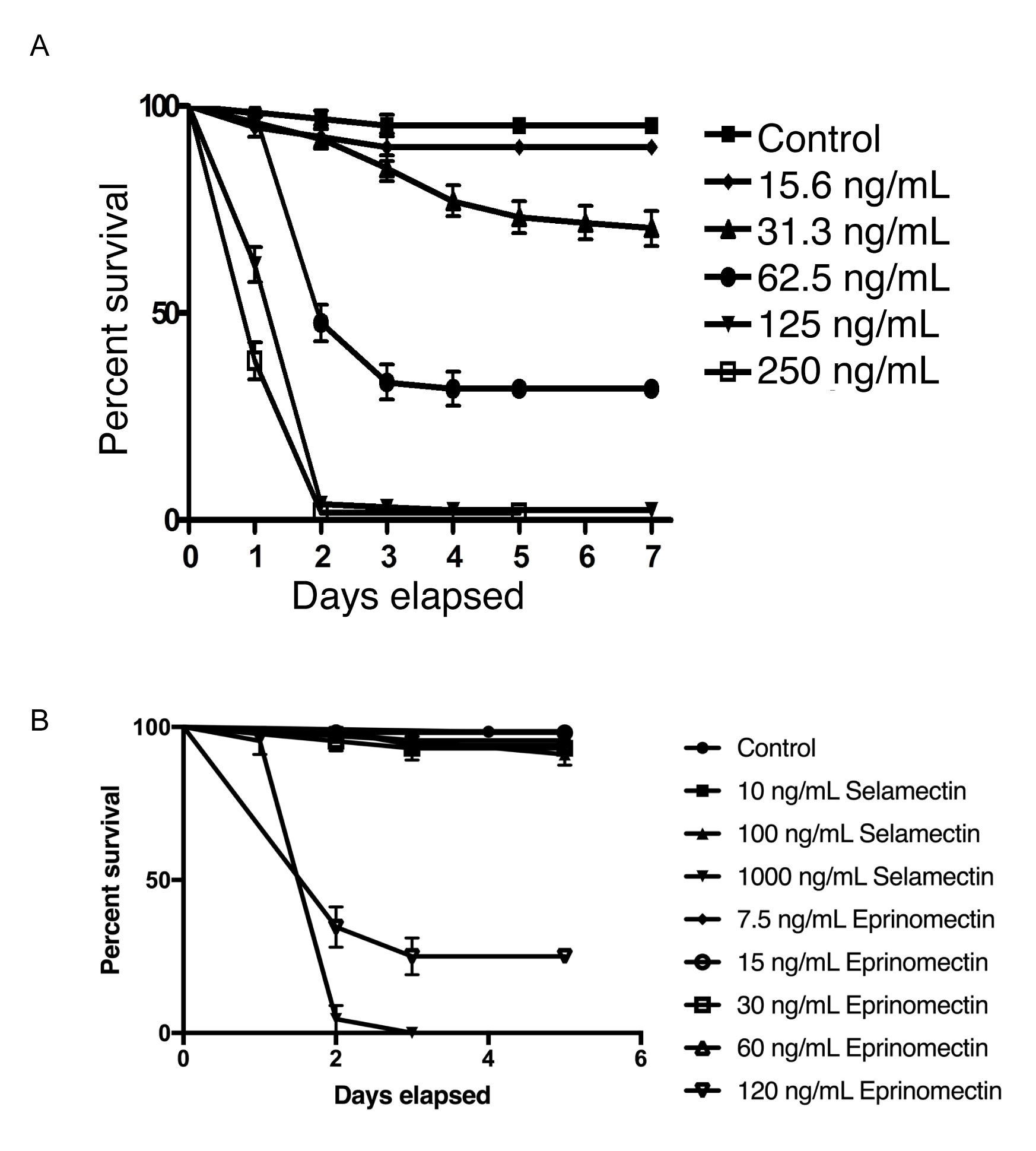

Supplement: S2 Fig — Cx. tarsalis mortality following blood feeding on IVM (A), selamectin (B), and eprinomectin (B). Cx. tarsalis were blood fed on increasing concentrations of endectocides and their mortality was observed to calculate lethal concentrations. Error bars indicate standard error. (TIF) [file pntd.0007210.s002.tif]

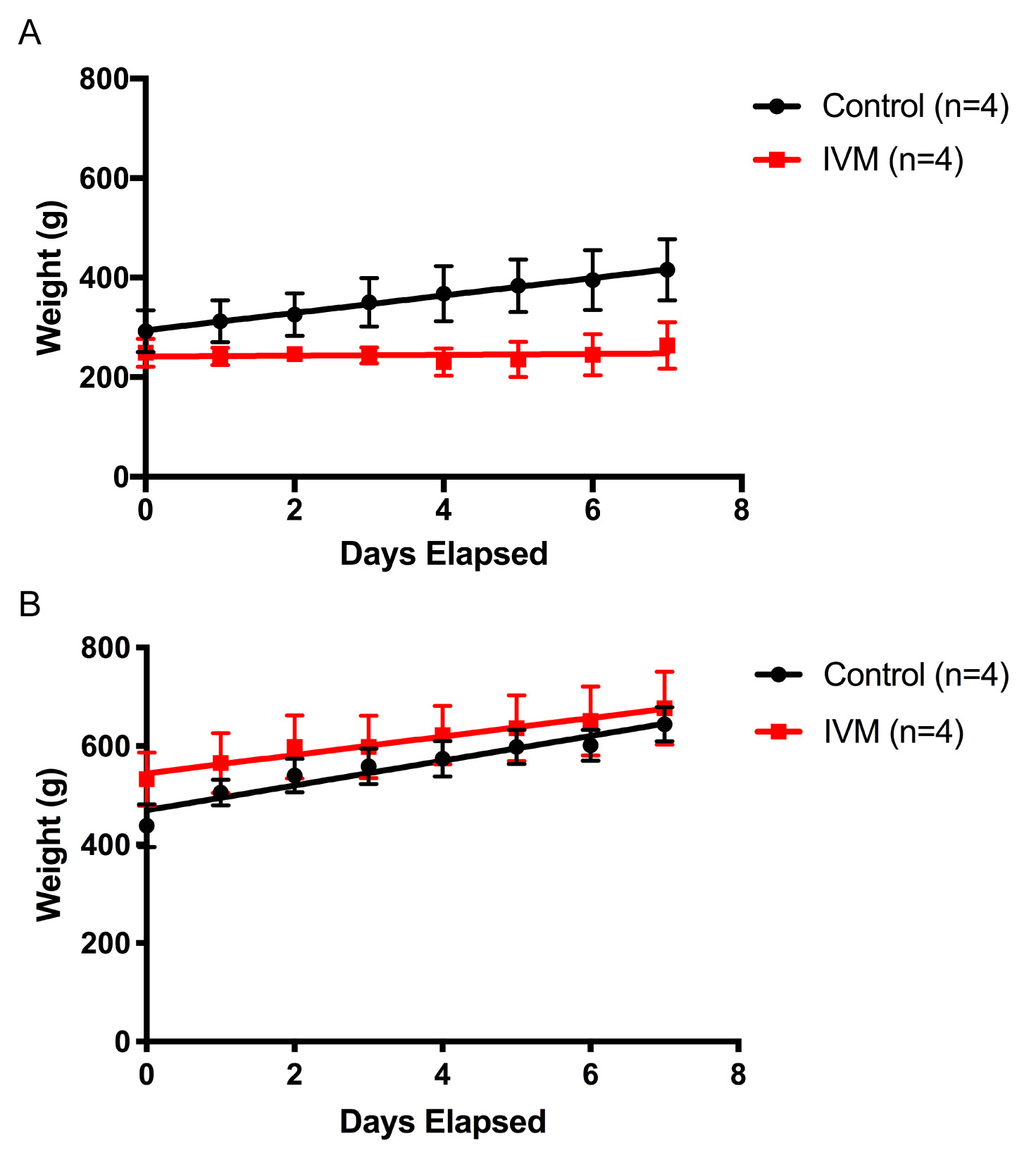

Supplement: S3 Fig — Linear relationship between chicken weight and days elapsed is shown where black lines indicate control groups and red lines indicate ivermectin-treated groups of chickens fed (A) Ivomec-formulation diet or (B) powder IVM-formulation diet. Error bars indicate standard deviation. (TIF) [file pntd.0007210.s003.tif]

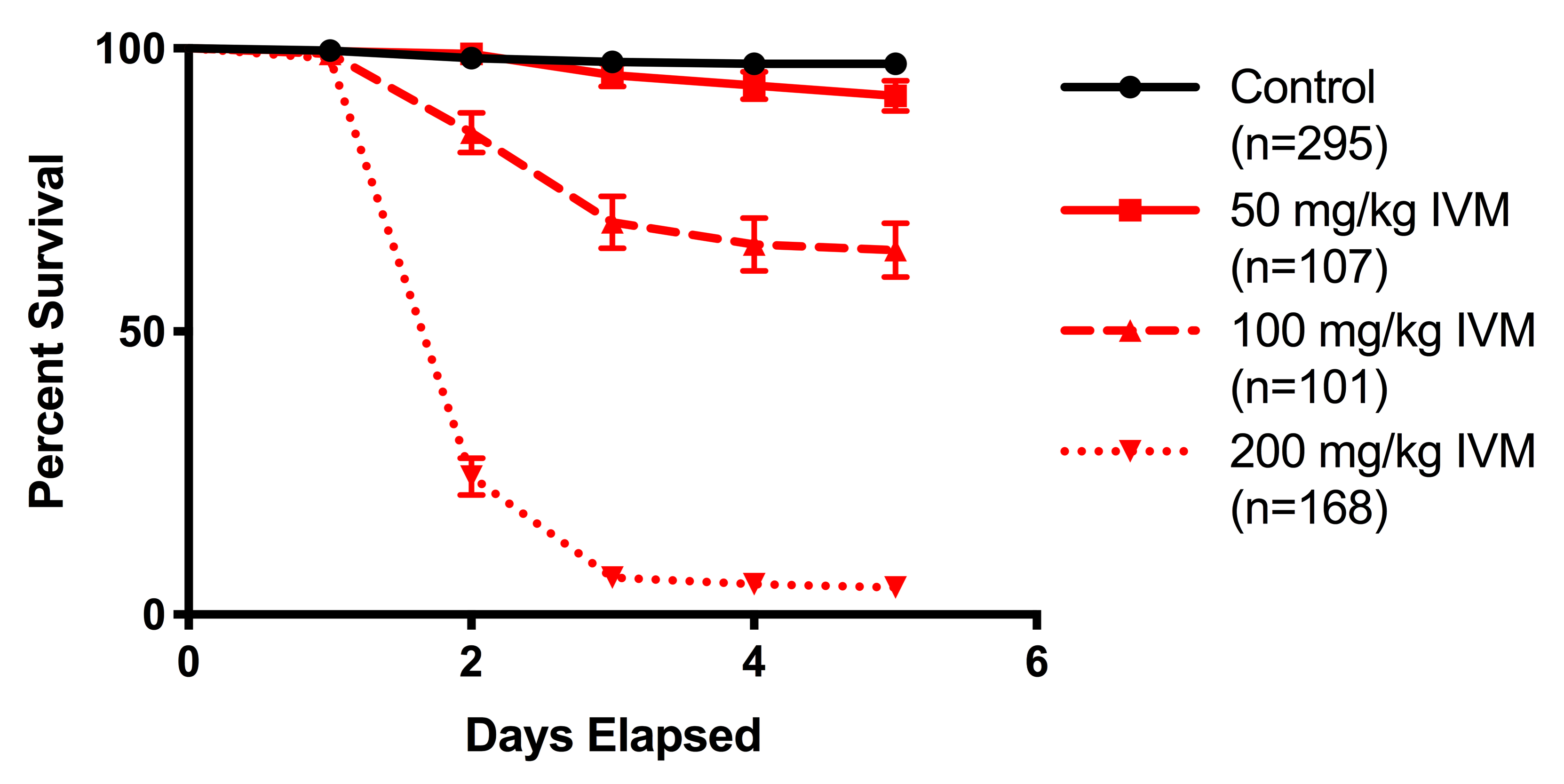

Supplement: S4 Fig — Cx. tarsalis survival following direct blood feeding on chickens that were fed Ivomec-formulation diet for 7 consecutive days at concentrations of 50 mg IVM/kg of diet, 100 mg IVM/kg of diet, and 200 mg IVM/kg of diet. Error bars indicate standard error. (TIF) [file pntd.0007210.s004.tif]

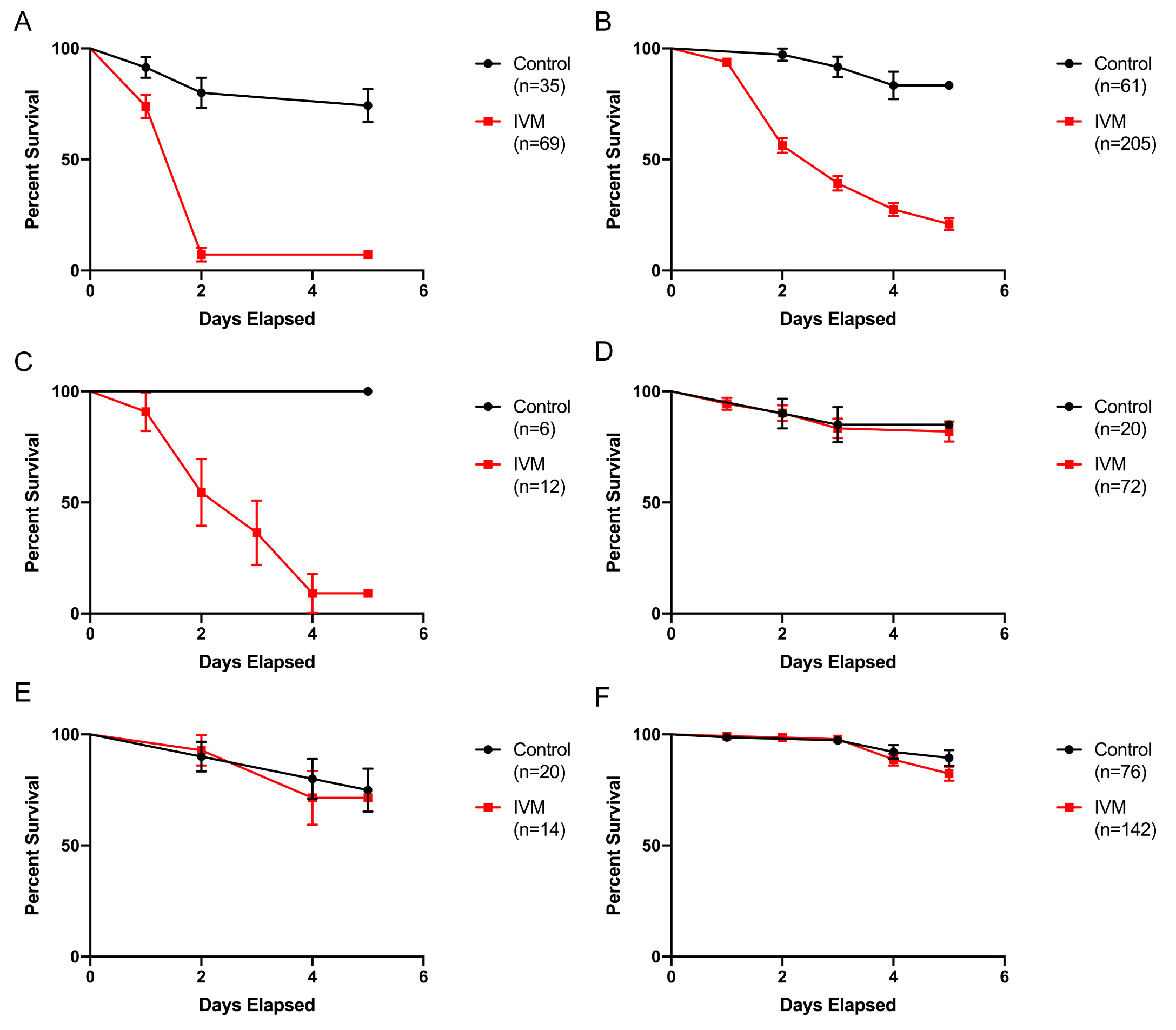

Supplement: S5 Fig — Cx. tarsalis survivorship following direct (left panels: A, C, E) or serum-replacement (right panels: B, D, F) blood feeding on chickens given powder-IVM diet at a concentration of 200 mg IVM/kg of diet for 7 days. (Top panels: A, B) Blood feeding occurred on, or using serum taken on, the last day treated diet was given to the IVM groups. (Middle panels: C, D) Blood feeding occurred on, or using serum taken on, the day after treated diet was withdrawn from the IVM groups. (Bottom panels: E, F) Blood feeding occurred on, or using serum taken on, the second day after treated diet was withdrawn from the IVM groups. Error bars indicate standard error. (TIF) [file pntd.0007210.s005.tif]
